# Monoclonal antibodies effectively potentiate complement activation and phagocytosis of *Staphylococcus epidermidis* in neonatal human plasma

**DOI:** 10.3389/fimmu.2022.933251

**Published:** 2022-07-29

**Authors:** Lisanne de Vor, Coco R. Beudeker, Anne Flier, Lisette M. Scheepmaker, Piet C. Aerts, Daniel C. Vijlbrief, Mireille N. Bekker, Frank J. Beurskens, Kok P. M. van Kessel, Carla J. C. de Haas, Suzan H. M. Rooijakkers, Michiel van der Flier

**Affiliations:** ^1^ Department of Medical Microbiology, University Medical Center Utrecht, Utrecht, Netherlands; ^2^ Department of Paediatric Infectious Diseases and Immunology, University Medical Center Utrecht, Utrecht, Netherlands; ^3^ Department of Neonatology, University Medical Center Utrecht, Utrecht, Netherlands; ^4^ Department of Obstetrics, University Medical Center Utrecht, Utrecht, Netherlands; ^5^ Genmab, Utrecht, Netherlands

**Keywords:** complement system, CLABSI, *Staphylococcus epidermidis*, monoclonal antibodies, phagocytosis, neonates

## Abstract

Central line associated bloodstream infections (CLABSI) with *Staphylococcus epidermidis* are a major cause of morbidity in neonates, who have an increased risk of infection because of their immature immune system. As especially preterm neonates suffer from antibody deficiency, clinical studies into preventive therapies have thus far focused on antibody supplementation with pooled intravenous immunoglobulins from healthy donors (IVIG) but with little success. Here we study the potential of monoclonal antibodies (mAbs) against *S. epidermidis* to induce phagocytic killing by human neutrophils. Nine different mAbs recognizing Staphylococcal surface components were cloned and expressed as human IgG1s. In binding assays, clones rF1, CR5133 and CR6453 showed the strongest binding to *S. epidermidis* ATCC14990 and CR5133 and CR6453 bound the majority of clinical isolates from neonatal sepsis (19 out of 20). To study the immune-activating potential of rF1, CR5133 and CR6453, bacteria were opsonized with mAbs in the presence or absence of complement. We observed that activation of the complement system is essential to induce efficient phagocytosis of *S. epidermidis*. Complement activation and phagocytic killing could be enhanced by Fc-mutations that improve IgG1 hexamerization on cellular surfaces. Finally, we studied the ability of the mAbs to activate complement in r-Hirudin neonatal plasma conditions. We show that classical pathway complement activity in plasma isolated from neonatal cord blood is comparable to adult levels. Furthermore, mAbs could greatly enhance phagocytosis of *S. epidermidis* in neonatal plasma. Altogether, our findings provide insights that are crucial for optimizing anti-*S. epidermidis* mAbs as prophylactic agents for neonatal CLABSI.

## Introduction

Neonatal sepsis is a major cause of mortality and morbidity (1, 2). Due to the use of indwelling medical devices, more than half of all late-onset sepsis episodes (occurring after more than 7 days of age) are caused by central line associated bloodstream infections (CLABSI) (3). The incidence of CLABSI is highest in preterm neonates (gestational age (GA) < 37 weeks) compared to term neonates (≥ 37 weeks GA) and children admitted to pediatric intensive care units (4–6). The most common pathogens found in CLABSI are coagulase negative staphylococci, with *Staphylococcus epidermidis* being the predominant species (7, 8). Currently, no effective strategy exists to prevent late-onset sepsis in neonates.

The high risk of CLABSI in neonates compared to older children is likely related to transient immunodeficiency of immaturity (9–15). Three elements that are crucial for immune protection against Gram-positive bacteria, namely neutrophils (16), antibodies and the complement system, have been reported to be impaired in neonates. First, neonates have low levels of circulating neutrophils (14). Neutrophils are highly specialized immune cells that circulate in the blood and are attracted to the site of infection to phagocytose bacteria. Following uptake by neutrophils, bacteria are subjected to high levels of reactive oxygen species (ROS) and degranulation of antimicrobial products that are destructive to staphylococci, this makes phagocytosis an efficient way to eliminate *S. epidermidis* (17, 18). Second, neonates have low levels of circulating antibodies. Because endogenous antibody synthesis only begins at birth, neonates depend on passive transfer of maternal antibodies over the placenta which mainly occurs in the final trimester of pregnancy. As a result, preterm neonates have low IgG levels (19). IgM and IgA are not transported over the placenta, thus all neonates newborns are IgM/IgA deficient at birth (9, 10). Third, the complement system is less active in neonates when compared to adults, but it can be activated in the presence of infection (11–13, 15). Both antibodies and complement concentrations in neonatal plasma increase with gestational age, meaning that extremely preterm neonates are more at risk than term neonates. Antibodies and complement components are important opsonins that are needed to label bacteria for efficient phagocytosis. Antibodies consist of two functional domains: the fragment antigen binding (Fab) region confers antigen specificity, while the crystallizable fragment (Fc) region drives interaction with other components of the immune system (20). After binding to the bacterial surface, IgG and IgA antibodies can directly induce phagocytosis *via* interaction with Fc receptors on neutrophils. IgG and IgM antibodies are both able to activate the classical pathway of the complement system which leads to deposition of C3b on the bacterial surface. C3b is recognized by complement receptors on neutrophils and leads to phagocytosis of the pathogen (21). Thus, antibodies play a central role in the immune response against Gram-positive bacteria such as *S. epidermidis.*

Clinical trials have assessed if antibody supplementation therapy with pooled intravenous immunoglobulins from healthy donors (IVIG) can ameliorate neonatal antibody deficiency and prevent or treat neonatal sepsis. These studies show only a 3% reduction in sepsis incidence in neonates and no improvement in mortality (22). We hypothesize that the disappointing efficacy of IVIG therapy in neonatal infections could be caused by low concentration of antibodies specific to the relevant neonatal pathogens.

In this study, we wondered whether pathogen specific monoclonal antibodies (mAbs), which have a sole specificity for one target, can be more effective than IVIG. Therefore, we cloned and expressed nine different mAbs recognizing staphylococcal surface components as human IgG1s. Of the three best binders (rF1, CR5133 and CR6453), we tested the ability to recognize a panel of 20 clinical *S. epidermidis* isolates from neonatal sepsis and showed that CR5133 and CR6453 bound to the majority (19 out of 20) of isolates. We then studied the immune activating potential of rF1-, CR5133- and CR6453-IgG1 and found that activation of the complement system is essential to induce efficient phagocytosis of *S. epidermidis*. As shown before on *Staphylococcus aureus* (23) and *Streptococcus pneumoniae* (24), phagocytosis and killing of *S. epidermidis* could be further enhanced by Fc-mutations that improve IgG hexamerization, which is needed for efficient activation of the classical pathway (25–27). Finally, we collected human cord blood from neonates to study the ability of the mAbs to activate the neonatal complement system. In contrast to what is reported (11–13, 15, 28), plasma isolated from preterm and term neonatal cord blood showed classical pathway complement activity comparable to adult levels. Furthermore, we demonstrate that pathogen specific monoclonal antibodies with hexamer enhancing mutations greatly enhanced phagocytosis of *S. epidermidis* in neonatal plasma by healthy donor neutrophils. Altogether, our findings provide insights that are crucial for optimizing anti-*S. epidermidis* mAbs as prophylactic or therapeutic agents for neonatal sepsis.

## Materials and methods

### Ethics statement

Human blood was obtained from healthy donors after informed consent was given by all subjects in accordance with the Declaration of Helsinki. Approval from the Medical Ethics Committee of the University Medical Center Utrecht was obtained (METC protocol 07-125/C approved on March 1, 2010). Cord blood (CB) was obtained from the umbilical cord after vaginal birth of neonates of different gestational age (GA); 32-37 weeks GA (n=2) and >37 weeks GA (n=3). Samples were collected at the obstetrics department and analyzed and stored anonymously. Written informed consent was obtained from the mother in accordance with the Declaration of Helsinki. The Ethics Committee for Biobanking of the University Medical Center Utrecht approved the collection protocol (TCBio 21/223, approved on June 14^th^, 2021).

### IgG and IgM depletion from human serum

Normal human serum (NHS) from twenty healthy donors was depleted of IgG and IgM as previously described (29). Briefly, affinity chromatography was used to capture IgG by using HiTrap protein G High Performance column (Cytiva, GE Healthcare) and IgM with Capture Select IgM Affinity Matrix (Thermo Fischer Scientific) from NHS. Complement levels and activity were determined after depletion, using enzyme-linked immunosorbent serological assay (ELISA) and classical/alternative pathway hemolytic assays. Although C1q is partially co-depleted during the procedure, C1q was not supplemented in IgG/IgM depleted NHS (ΔNHS), because supplementation with 70 µg/ml C1q did not alter complement deposition on *S. epidermidis* **(** **Figure S1** **)**, indicating that the amount of C1q left was sufficient.

### Generation of human monoclonal antibodies

IgG1 mAbs A120, CR5133, CR6166 and CR6176 were produced by Genmab (Utrecht, the Netherlands) as described previously [WO 2017/198731 Al (30)]. IgG1 clones M130, CR5132, CR6171, CR6453, rF1 and G-2A2 (aDNP) and the subtypes of rF1, CR5133, CR6453 and G-2A2 (IgG3, IgG1 E345K, IgG3 E345K) were produced as described previously (23, 31). For mAb expression, variable heavy (VH) chain and variable light (VL) chain sequences were cloned in expression vectors pcDNA3.3 (Thermo Fisher Scientific) as described in Vink et al. (30) or pcDNA3.4 (Thermo Fisher Scientific) as described in de Vor et al. (31), containing human IgG1 heavy chain (HC) and light chain (LC) constant regions as indicated in **Table S1**. The E345K single mutation of Glu at position 345 into Lys was introduced in the heavy chain expression vectors by gene synthesis (IDT (Integrated DNA Technologies)). Variable heavy (VH) and light chain (VL) sequences from all antibodies were obtained from scientific publications or patents (**Table S1**). Antibodies were expressed as IgG1,κ except for CR6166 which was expressed as IgG1,λ. Plasmid DNA mixtures encoding both heavy and light chains of antibodies were transiently transfected in EXPI293F (Life technologies, USA) as described before (30) (31). IgG1 antibodies were isolated using HiTrap Protein A High Performance column (Cytiva, GE Healthcare) and IgG3 antibodies were collected using HiTrap Protein G High Performance column (Cytiva, GE Healthcare) in the Äkta Pure protein chromatography system (GE Healthcare). All antibody fractions were dialyzed overnight in PBS and filter-sterilized through 0.22 µm SpinX-filters. Size exclusion chromatography (SEC) (Cytiva, GE Healthcare) was performed to check for mAb homogeneity, and the monomeric fraction was separated when aggregation levels exceeded 5%. Final antibody concentration was determined by measuring the absorbance at 280 nm and antibodies were stored at -80°C or 4°C.

### Bacterial strains and growth conditions

*S. epidermidis* type strain ATCC 14990 (32, 33) and twenty clinical bacteremia isolates from neonates admitted to the Wilhelmina Children’s hospital (UMC Utrecht) were used in this study. Strains were grown overnight at 37°C on sheep blood agar (SBA) and were cultured overnight in Tryptic Soy Broth (TSB). Next, they were subcultured and grown to exponential phase for ± 2.5 hours. For flow cytometry, exponential phase bacteria were washed in PBS and incubated in 250 µg/ml fluorescein isothiocyanate (FITC) (Sigma Aldrich) in PBS for 30 minutes at 4°C. After washing, FITC labeled bacteria were diluted in RPMI (Gibco) supplemented with 0.05% human serum albumin (HSA) (Sanquin products) and the bacterial concentration was determined by flow cytometry using a MACSQuant analyzer (Miltenyi), counting bacterial events in a defined volume. Bacteria were aliquoted and stored at -20°C. When indicated, freshly grown exponential cultures were used of which the concentration was determined based on optical density at 600 nm (OD_600_) (OD 1.0 = 5 x 10^8 bact/ml).

### Antibody binding to *S. epidermidis*

For the initial screening of mAb binding to *S. epidermidis*, freshly grown bacteria were washed and diluted in PBS supplemented with 0.1% bovine serum albumin (BSA) (Serva). For all other binding experiments, FITC labelled 14990 and N2297 were used. A total of 150,000 bacteria were incubated with a concentration range of mAbs or IVIG (Nanogam, Sanquin) in PBS-BSA in a round-bottom 96-wells plate for 30 minutes at 4°C, shaking (± 750 rpm). After washing, bacteria were further incubated for 30 minutes at 4°C, shaking (± 750 rpm) with 30 µl APC-conjugated goat anti-human IgG (H+L) F(ab’)_2_ antibody (Jackson Immunoresearch, 1 µg/ml) or APC-conjugated donkey anti-human IgG (H+L) F(ab’)_2_ antibody (Jackson Immunoresearch, 1:250) to detect both lambda and kappa chains or AlexaFluor647(AF647)-conjugated goat anti-human kappa F(‘ab)_2_ antibody (Southern Biotech, 1 µg/ml) to compare binding of IgG1, IgG1 E345K, IgG3 and IgG3 E345K. After incubation with detection antibodies, bacteria were washed and fixed in 1% paraformaldehyde (PFA) (Polysciences) in PBS. Samples were measured by flow cytometry (BD FACSVerse), and data analyzed by FlowJo Software (Version 10). A control sample was used to set FSC-SSC gates to exclude debris and large aggregates of bacteria. In case we used FITC labelled bacteria, only FITC-positive events were analyzed. Data are presented as APC or AF647 geometric mean fluorescence intensity (GeoMFI ± SD).

### Phagocytosis of *S. epidermidis* by neutrophils

Human neutrophils were isolated from blood of healthy donors by Ficoll-Histopaque gradient centrifugation and suspended in RPMI-HSA (34). For opsonization, 750,000 FITC labelled bacteria (20 µL) were incubated with 10 µL mAbs or IVIG supplemented with 10 µL ΔNHS or neonatal plasma (final concentration 1%) or buffer, to investigate complement- or Fc-receptor mediated phagocytosis, respectively. Bacteria were incubated in a round-bottom 96-wells plate for 15 minutes, at 37°C, shaking (± 750 rpm). Subsequently, 75,000 neutrophils (10 µL) were added, giving a 10:1 bacteria-to-cell ratio and samples were incubated for 15 minutes at 37°C, shaking (± 750 rpm). The reaction was stopped with 1% ice cold PFA in RPMI-HSA and FITC fluorescence was measured by flow cytometry (BD FACSVerse). To exclude debris, a neutrophil population incubated in RPMI-HSA alone was used to set FSC-SSC gates. Data were analyzed by FlowJo (version 10) and presented as FITC GeoMFI ± SD of the neutrophil population.

### Killing of *S. epidermidis*

Freshly grown, exponential phase *S. epidermidis* was washed with Hanks Balanced Salt Solution (HBSS) (BioWitthaker) supplemented with 0.1% HSA. For opsonization, 850,000 bacteria were incubated with NHS or mAbs and ΔNHS in HBSS-HSA (concentrations indicated in figure legends) in a round bottom 96-deep-well plate for 30 minutes at 37°C, shaking (± 750 rpm) (final volume: 15 µl). After 30 minutes opsonization, 850,000 neutrophils were added (MOI 1:1) and further incubated for an additional 90 minutes at 37°C, 5% CO_2_ with open cover, shaking (± 750 rpm) (final volume: 100 µl). The reaction was stopped by adding 900 µl of ice-cold, freshly prepared, 0.2 µm sterile filtered 0.3% saponin (w/v, Sigma) in Milli-Q water. Samples were incubated for 5-15 minutes at 4°C, shaking (± 750 rpm). Total viable bacterial counts were determined by serial dilution and plating. Data are presented as CFU ± SD. The following control conditions were always included: 10% NHS with neutrophils (positive control for killing), heat-inactivated-ΔNHS with neutrophils (negative control for killing), HBSS-HSA with neutrophils only (sterility control) and HBSS-HSA with bacteria only (reference control for the amount of bacteria added).

### Production of anti-C3b AF647 conjugate

2000 µg/ml mouse IgG2a mAb against a neo-epitope C3b (clone Bh6) (35) was incubated with AF647 NHS ester (Life Technologies) (final concentration: 100 µg/ml) and sodium carbonate (pH 9.4, final concentration: 0.1 M) for 70 minutes, room temperature, rotating. The sample was separated from aggregates by spinning through 0.22 µm SpinX filter columns (Corning life Sciences BV) for 2 minutes at 1500x *g*. To remove free AF647 from the mixture, filtrate was transferred to a sterile PBS washed Zeba spin column (Thermo Scientific) and spun for 2 minutes at 1500x *g*. From OD_280_ and OD_650_ Nanodrop (Thermo Scientific) measurements, the degree of labelling (DOL) was determined at 4.14. The conjugate was checked for integrity by SDS-PAGE and fluorescence of antibody-conjugate was confirmed using ImageQuant LAS 4000.

### Complement deposition on *S. epidermidis*

To determine C3b deposition on *S. epidermidis*, 150,000 FITC labeled bacteria were incubated with mAbs supplemented with 1% ΔNHS in RPMI-HSA for 30 minutes at 37°C, shaking (± 750 rpm) (final volume: 30 µl). After washing twice with PBS-BSA, bacteria were further incubated for an additional 30 minutes at 4°C, shaking (± 750 rpm) with 15 µl anti-C3b AF647 detection antibody (3 µg/ml) in PBS-BSA. Bacteria were washed with PBS-BSA and fixed in 1% PFA in PBS-BSA for at least 30 minutes at 4°C, shaking (± 750 rpm) after which fluorescence was measured by flow cytometry (BD FACSVerse). To exclude debris and large aggregates of bacteria, control bacteria incubated in RPMI-HSA alone were used to set FSC-SSC gates, only FITC-positive events were analyzed. Data were analyzed by FlowJo Software (Version 10) and presented as AF647 GeoMFI ± SD.

### Collection of plasma samples

Cord blood or peripheral venous blood of healthy human donors was collected in S-Monovette r-Hirudin tubes (Sarstedt) to preserve complement activity (36). The blood was centrifuged at 1000x g for 5 minutes and plasma was removed and stored immediately at -80°C. Plasma of 27 healthy donors was pooled before freezing and cord blood plasma was stored individually. For complement inactivation, the plasma was heat-inactivated for 30 minutes at 56°C.

### Complement ELISAs

Complement ELISAs were performed as previously described (37) with minor modifications. Briefly, microtiter plates (Nunc maxisorp) were pre-coated with 3 µg/mL IgM (Sigma) for classical pathway (CP) ELISA, with 20 µg/ml LPS (from *Salmonella enteriditis*, Sigma) for alternative pathway (AP) ELISA and with 20 µg/mL mannan (from *Saccharomyces cerevisiae*, Sigma) for lectin pathway (LP) ELISA. All coatings were prepared in 0.1 M carbonate buffer (pH 9) and incubated overnight at room temp. Plasma samples were diluted in Veronal Buffered Saline (VBS) + 0.1% gelatin + 5 mM MgCl_2_ + 10 mM EGTA for AP ELISA and in VBS + 0.1% gelatin + 0.5 mM CaCl_2_ + 0.25 mM MgCl_2_ for CP ELISA and LP ELISA. After washing the plates with PBS 0.05% Tween-20, wells were blocked with 4% BSA in PBS-Tween. Then, plasma samples were added for 1 hour at 37°C in the appropriate buffer and dilutions. After additional washing, deposited C3b was detected using DIG-labelled mouse anti C3 antibody (WM1, 0.1 µg/mL) followed by Peroxidase conjugated sheep anti-DIG-Fab fragments (1:8000, Roche 11207733910). Finally, the plates were washed and developed using 3,3’,5,5’-tetramethylbenzidine (Thermo Fisher). The reaction was stopped by addition of 1 N H_2_SO_4_. Absorption at 450nm was measured using a microplate reader (Biorad).

### Statistical analysis

Statistical analyses were performed in Prism software (version 8.3; Graphpad). The tests used to calculate P-values are indicated in the figure legends. The number of independent biological repeats per graph is indicated in the figure legends.

## Results

### Production and identification of human monoclonal antibodies against *S. epidermidis*

First, we produced a panel of nine mAbs by cloning the variable light chain (VL) and heavy chain (VH) sequences derived from scientific publications or patents (**Table S1**) into expression vectors to produce full-length human IgG1 antibodies. We selected clones rF1 (binding surface proteins of the SDR family (38)), M130 (binding peptidoglycan [US20030228322A]), A120 (binding LTA [WO-03059260-A3]), CR5132 (possible target LTA [2012/0141493 A1] or Wall Teichoic Acid (31), CR5133 (possible target LTA [2012/0141493 A1]) and CR6453 (possible target LTA [2012/0141493 A1]) that have already been described to bind and induce opsonophagocytic killing of different *S. epidermidis* strains, but not to our model strain ATCC 14990 (33). We also included clones CR6166, CR6171 and CR6176, that are related to CR6453 and were screened for cross-reactivity because they bind *S. aureus* [2012/0141493 A1]. As a negative control, we produced one antibody recognizing the hapten dinitrophenol (DNP) (G2a2-IgG1) (39).

**Figure 1:**
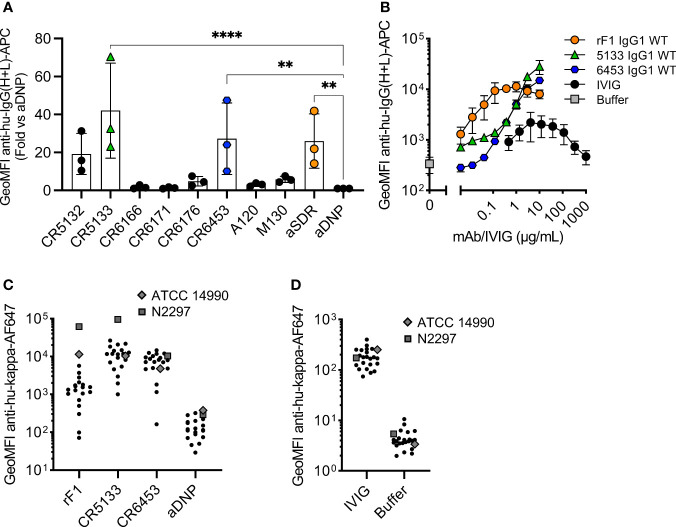
Identifying mAbs that bind *S. epidermidis* neonate isolates. **(A)** Screening mAb binding to *S. epidermidis* ATCC 14990. Bacteria were incubated with 2 µg/mL IgG1. MAb binding was detected using goat anti-hu-IgG(H+L)-APC and using flow cytometry. Data represent GeoMFI ± SD normalized to aDNP (ctrl)-IgG1 of three independent experiments. One-way ANOVA followed by Dunnett test was performed to test for differences in antibody binding versus aDNP and displayed only when significant as **P ≤ 0.01 and ****P ≤ 0.0001. **(B)** Titration of binding mAbs. FITC labelled ATCC 14990 were incubated in a 3-fold dilution range from 1000 µg/mL for IVIG and from 10 µg/mL for rF1-IgG1, CR5133-IgG1 or CR6453-IgG1. MAb binding detected with donkey anti-hu-IgG(H+L)-APC and analysed with flow cytometry. Data represent GeoMFI ± SD of three independent experiments. Histograms of flow cytometry analysis are included in **Figure S2**. **(C)** IVIG binding to clinical isolates. 20 clinical isolates and ATCC 14990, were incubated with 25 µg/mL IVIG. IVIG binding was detected with goat anti-hu-kappa-AF647. Data points are represented as mean AF647 GeoMFI of one experiment. **(D)** mAb binding to clinical isolates. 20 clinical isolates and ATCC 14990, were incubated with 2 µg/mL nM rF1-, CR5133-, CR6453- and aDNP-IgG1. mAb binding was detected with goat anti-hu-kappa-AF647. Data points are represented as mean AF647 GeoMFI of two independent experiments.

Next, we compared the binding of the panel of IgG1 mAbs to the sequenced common laboratory strain *S. epidermidis* ATCC 14990 (33), using flow cytometry. Compared to the negative control antibody (aDNP-IgG1) which does not recognize a bacterial component, only three antibodies (CR5133-IgG1, CR6453-IgG1 and rF1-IgG1) significantly bound to ATCC 14990 (**Figure 1A**). Although binding to other *S. epidermidis* strains has been described, we could not detect significant binding of IgG1 mAbs M130 and A120 to ATCC 14990. Our results indicate that CR5132 also specifically binds ATCC14490, although not significant. As CR5132 has been described to bind the same target as CR5133, which does show stronger and significant binding, we selected CR5133 instead of CR5132 for further characterization. After titration, we detected a higher binding signal at lower concentrations for rF1-IgG1 compared to CR5133- and CR6453-IgG1 **(** **Figure 1B**), indicating that rF1-IgG1 is the best binding mAb in the panel.

We also compared the strength and broad specificity of mAb binding and IVIG binding to *S. epidermidis*. We could measure binding of IVIG antibodies to ATCC 14990, but this required higher concentrations (10 µg/mL IVIG *vs* 0.004 µg/mL rF1-IgG1 or 0.37 µg/mL CR5133- and CR6453-IgG1) to reach a similar binding level. One advantage of the polyclonal nature of IVIG is that it may be possible to target a broad range of isolates. As mAbs recognize one unique target, it is important that this target is present on the majority of *S. epidermidis* isolates found in the clinic. To test the broad specificity of rF1-, CR5133- and CR6453-IgG1 when compared to IVIG, we collected a set of twenty clinical *S. epidermidis* isolates from neonatal sepsis cases. Indeed, we could detect binding of IVIG to all clinical isolates in the panel (**Figure 1C**). CR5133- and CR6453-IgG1 bound 19/20 isolates and rF1 bound 11/20 isolates with good capacity (classified as at least 10x binding compared to ctrl-IgG1) (**Figure 1D** **)**. Thus, although CR5133- and CR6453-IgG1 bind less well to ATCC 14990, overall they bind a larger fraction of clinical isolates compared to rF1-IgG1.

### Activation of the complement system greatly enhances phagocytosis by mAbs

**Figure 2:**
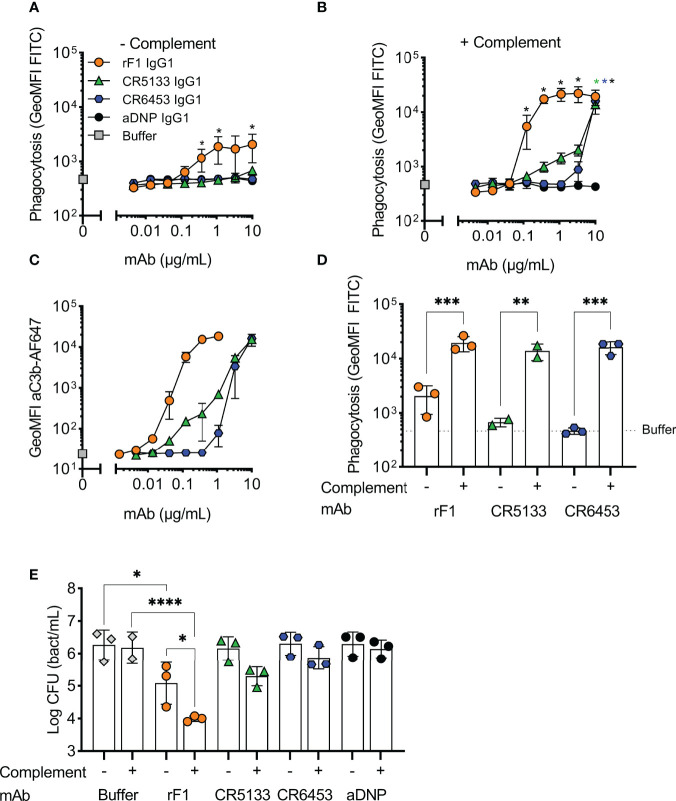
Activation of the complement system greatly enhances phagocytosis and killing with mAbs. **(A,B)** Phagocytosis of *S. epidermidis* ATCC 14990 by human neutrophils ((t=15 min), MOI 10:1) in **(A)** absence or **(B)** presence of complement. FITC labelled bacteria were incubated in **(A)** RPMI-HSA or **(B)** 1% IgG/IgM-depleted normal human serum (ΔNHS) supplemented with a concentration range of rF1, CR5133, CR6453, aDNP IgG1 or buffer. Phagocytosis was quantified by flow cytometry and plotted as FITC GeoMFI of the neutrophils population. The gating strategy at 1 µg/mL mAb is shown in **Figure S3**. Data represent mean ± SD of three independent experiments. One-way ANOVA followed by Bonferroni correction was used to test the effect of mAb addition compared to aDNP-IgG1. Test results were displayed only when significant as *P ≤ 0.05 (black for rF1, green for CR5133, blue for CR6453). **(C)** C3b deposition by mAbs. FITC labelled *S. epidermidis* ATCC 14990 were incubated in 1% ΔNHS supplemented with a concentration range of mAb. C3b deposition was detected by flow cytometry using an anti-neoC3b-AF647 antibody conjugate and plotted as AF647 GeoMFI of the FITC+ve bacterial population. Data represent mean ± SD of three independent experiments. **(D)** Comparison of mAb (10 µg/mL) induced phagocytosis in absence and presence of complement. One-way ANOVA was performed to test for the effect of complement addition on phagocytosis and displayed as *P ≤ 0.05, **P ≤ 0.01, ***P ≤ 0.001 and ****P ≤ 0.0001. **(E)** Killing of *S. epidermidis* ATCC 14990 by human neutrophils (t=90 min, MOI 1:1) in absence or presence of complement. Bacteria were incubated in 10% heat-inactivated (HI)-ΔNHS (–) or 10% ΔNHS (+) supplemented with buffer (dashed horizontal line) or 14.8 µg/mL (100 nM) rF1-, CR5133-, CR6453- or aDNP-IgG1. Data represent mean ± SD of three independent experiments. One-way ANOVA followed by Bonferroni correction was used to test the effect of mAb addition to HI-ΔNHS, mAb addition to ΔNHS and to test the effect of complement addition for each mAb specifically. Test results were displayed only when significant as *P ≤ 0.05, **P ≤ 0.01, ***P ≤ 0.001 and ****P ≤ 0.0001.

We then evaluated whether the selected IgG1 mAbs could induce phagocytosis of *S. epidermidis* by human neutrophils. First, we studied their capacity to directly engage Fc gamma receptors in the absence of the complement system. To study this, we incubated freshly isolated neutrophils together with *S. epidermidis* ATCC 14990 opsonized with mAb at a multiplicity of infection (MOI) of 10:1. CR5133-IgG1, CR6453-IgG1 and the negative control aDNP-IgG1 could not induce Fc gamma mediated phagocytosis after 15 minutes of co-incubation (**Figure 2A**). Only rF1-IgG1 was capable of inducing phagocytosis in absence of the complement system. When 1% normal human serum depleted of IgG and IgM (ΔNHS) as complement source was added, phagocytosis was enhanced (**Figure 2B**), with rF1-IgG1 reaching high phagocytosis levels from a concentration above 0.1 µg/mL. Phagocytosis induced by CR5133-IgG1 and CR6453-IgG1 was also enhanced, but these antibodies were less efficient than rF1-IgG1 because higher concentrations were needed to reach similar phagocytosis levels as rF1-IgG1. This observation was very consistent with the ability of the mAbs to induce complement deposition on the bacterial surface, measured using a fluorescent mAb recognizing a neoepitope in C3b (35) and flow cytometry (**Figure 2C**). RF1-IgG1 was the most efficient, followed by CR5133-IgG1 and CR6453-IgG1. At a fixed concentration of 10 µg/mL, we observed large differences between phagocytosis induced in absence or presence of complement, showing that mAb binding and the complement system are essential for efficient phagocytosis (**Figure 2D**). Finally, we tested the ability of mAbs to induce phagocytic killing, measured as a reduction in colony forming units (CFU) after prolonged incubation (90 minutes) with neutrophils at an MOI of 1:1. Consistent with the phagocytosis data showing that only rF1-IgG1 can induce Fc-mediated phagocytosis (**Figure 2A**), we observed that rF1-IgG1, but not CR5133-IgG1 and CR6453-IgG1, could induce phagocytic killing in absence of complement (**Figure 2E**). Also, in the presence of complement, killing by rF1-IgG1 was more efficient than in the absence of complement. CR5133-IgG1 and CR6453-IgG1 seemed to perform better in the presence of complement, although no significant killing compared to buffer treated samples was observed (**Figure 2E**). Overall, there are potent mAbs (rF1-IgG1) and less potent mAbs (CR5133- and CR6453-IgG1), and complement enhances phagocytic uptake and killing.

### Hexamer-enhancing mutations in CR5133- and CR6453-IgG1 enhance phagocytosis and killing of *S. epidermidis*

**Figure 3:**
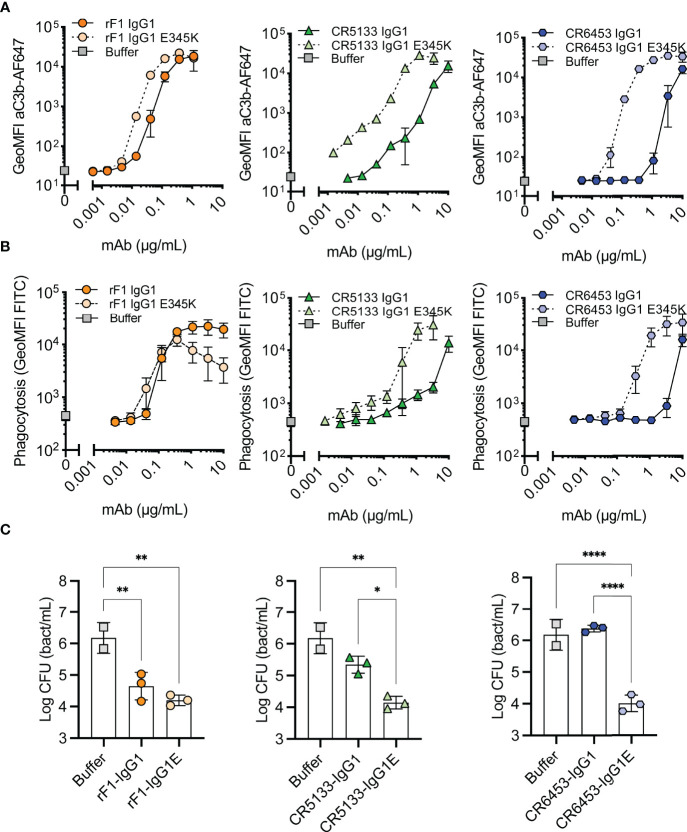
Hexamer enhancing mutations significantly improve effector functions against *S. epidermidis.* **(A)** Effect of introduced hexamer-enhancing mutations on deposition of C3b. FITC labelled *S. epidermidis* ATCC 14990 were incubated in 1% ΔNHS supplemented with a concentration range of mAb in IgG1 or IgG1-E345K variant. C3b deposition was detected by flow cytometry using an anti-neoC3b-AF647 antibody conjugate and plotted as AF647 GeoMFI of the FITC+ve bacterial population. Data represent mean ± SD of three independent experiments. **(B)** Phagocytosis of *S. epidermidis* ATCC 14990 by neutrophils (MOI 10:1) in the presence of complement. Bacteria were prepared as in **(A)**. Phagocytosis was assessed by flow cytometry and plotted as FITC GeoMFI of the neutrophils population. Data represent mean ± SD of three independent experiments. Data shown for IgG1 are identical to data shown in **Figure 2B**. **(C)** Killing of *S. epidermidis* ATCC 14990 by neutrophils (MOI 1:1) in presence of complement. Bacteria were incubated in 10% ΔNHS supplemented with 0.148µg/mL (1nM) rF1, 1.48µg/mL (10nM) CR5133 or CR6453. All concentrations tested can be viewed in **Figure S5**. Bacterial survival was quantified after neutrophils lysis by serial dilution and CFU counting. Data represent mean ± SD of three independent experiments. One-way ANOVA followed by Bonferroni correction was used to test the effect of mAb addition in ΔNHS, as well as the difference in bacterial survival of WT *vs* hexabody, *P ≤ 0.05, **P ≤ 0.01 and ****P ≤ 0.0001.

For optimal interaction with the six globular headpieces of C1q, target bound IgG molecules require organization into higher-order oligomers (IgG hexamers), which occurs *via* noncovalent Fc-Fc interactions (26, 27). Therefore, we hypothesized that hexamer-enhancing mutations can improve complement activation and phagocytosis of *S. epidermidis*. We introduced mutation E345K in the Fc backbone of rF1-IgG1, CR5133-IgG1, CR6453-IgG1. This mutation was selected based on previous results obtained with *Streptococcus pneumoniae* (24). After confirming that introduction of the E345K-mutations did not affect antibody binding to *S. epidermidis* (**Figure S4**), we tested their ability to induce C3b deposition (**Figure 3A**), phagocytosis (**Figure 3B**) and killing (**Figure 3C**). Introduction of the hexamer-enhancing mutation into the already potent rF1-IgG1 could only slightly improve complement deposition (**Figure 3A**). In contrast, hexamer-enhanced variants of CR5133-IgG1 and CR6453-IgG1 induced very potent complement deposition compared to the WT IgG1 mAbs. For CR5133-IgG1 and CR6453-IgG1, increased complement deposition by the IgG1-E345K variants translated into more efficient phagocytosis (**Figure 3B**), while for rF1-IgG1 we only measured a difference in phagocytosis at the lowest mAb concentrations. Interestingly, rF1-IgG1E E345K at higher concentrations induced less phagocytosis than rF1-IgG1, which did not correlate to C3b deposition. In general, more phagocytosis benefit was gained by alteration of CR5133- and CR6453-IgG1 than by alteration of the already potent rF1-IgG1, shifting the effectiveness to a lower mAb concentration. Finally, we determined the effect of the hexamer-enhancing mutation on killing. At the lowest concentration tested (0.147 µg/mL), no additive effects of rF1-IgG1 E345K compared to rF1-IgG1 were observed (**Figure 3C**), presumably because incubation with 0.147 µg/mL rF1-IgG1 already reached the maximum killing capacity of the assay. In line with this hypothesis, the use of higher concentrations rF1-IgG1 or rF1-IgG1 E345K did not increase killing (**Figure S5A**). However, we observed a striking increase in killing after introducing the hexamer-enhancing mutation in CR5133- and especially in CR6453-IgG1 (**Figure 3C**). Again, these data show that rF1-IgG1 is a potent mAb that could not be improved further by introducing a hexamer-enhancing mutation. Importantly, our data also shows that less potent IgG1 mAbs, such as CR5133 and CR6453, can be greatly improved by introducing hexamer-enhancing mutations.

Because antibodies of subclass IgG3 bind C1q more stable than IgG1 (40), we also studied the effect of subclass switching to IgG3. As the molecular weight of IgG3 is slightly different from the molecular weight of IgG1, we compared concentrations in nM instead of µg/mL. Subclass switching of rF1-IgG1 to IgG3 did not change its killing capacity (**Figure S5A**), again presumably because the maximum killing capacity was already reached by the IgG1 variant. For mAbs CR5133 and CR6453, the effect of subclass switching to IgG3 was only detectable at a concentration that was 10 times higher (100 nM) than the concentration at which the effect of hexamer-enhancing mutations was detectable (10 nM) (**Figure S5BC**). This indicates that introducing the E345K mutation is more effective than subclass switching to IgG3. Moreover, introduction of the hexamer-enhancing mutation in the IgG3 subclass (IgG3E) did not improve killing beyond its IgG1E counterpart.

### Plasma isolated from human umbilical cord blood retains classical pathway activity

**Figure 4:**
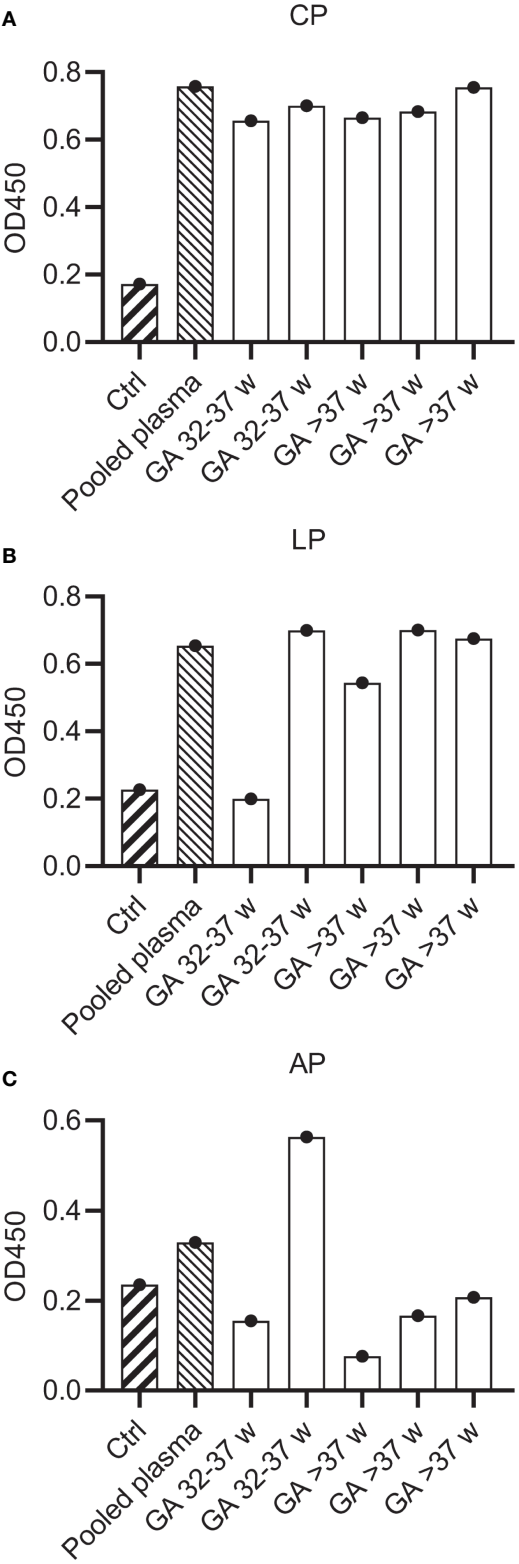
Complement activity of neonatal plasma. Complement activity in pooled human hiridun plasma and neonatal hirudin plasma (n=5) was determined in complement ELISAs, detecting deposition of C3b. Plates were coated with **(A)** IgM and 2% plasma to determine CP activity, **(B)** mannan and 2% plasma to determine LP activity and **(C)** LPS and 30% plasma to determine AP activity. Ctrl wells were uncoated and incubated with pooled adult plasma. CP, Classical pathway; LP, Lectin pathway; AP, Alternative pathway.

Previous studies have reported that the neonatal complement system is less active than in healthy adults (11–13, 15). To investigate if mAbs can also activate the neonatal complement system, we first compared neonatal cord blood plasma samples to adult pooled human plasma in complement activity ELISAs to measure classical (CP), lectin (LP) and alternative (AP) pathway activity. We collected cord blood plasma from neonates (n=5) with a gestational age ranging from 32-42 weeks. Parallel, plasma from healthy adult volunteers was collected in the exact same procedure. To preserve complement activity, all samples were collected in r-Hirudin tubes. Hirudin is a direct thrombin inhibitor, which does not interfere with the complement system, in contrast to other anticoagulants such as heparin or sodium citrate (41, 42). We showed that in all neonatal samples the CP was equally active to adult pooled human plasma (**Figure 4A**). The LP was decreased in activity compared to pooled human plasma in only one neonatal donor (**Figure 4B**). On the other hand, the alternative pathway was decreased in all but one neonatal donor (**Figure 4C**). Thus, although not all complement pathways are equally active in neonatal plasma, we showed that the classical pathway, which is activated by mAbs, is not impaired.

### Anti-*S. epidermidis* mAbs react with the neonatal complement system

Finally, we used the same assay to study if neonatal plasma could react with mAbs to opsonize bacteria for phagocytosis. Because umbilical cord blood was collected anonymously, there is a possibility that mothers received prophylaxis against group B streptococci by Amoxicillin/Clavulanic acid (Augmentin^®^) during labor, which is transferred to the neonatal plasma. Unfortunately, ATCC 14990 was sensitive to this antibiotic. As this could interfere with our assays, we selected a clinical isolate (N2297) that was resistant to Amoxicillin/Clavulanic acid for use in the assays with neonatal plasma. First, we confirmed that all mAbs can bind N2297 and that introduction of the E-mutation did not affect mAb binding (**Figure S6**). We also compared mAb and IVIG binding and again observed that all mAbs showed increased binding compared to IVIG, even at very low concentrations.

**Figure 5:**
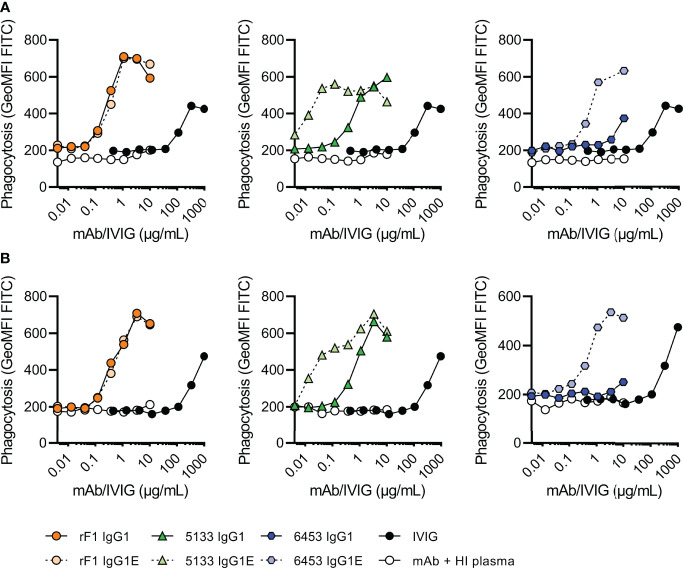
Phagocytosis of FITC labelled N2297 after incubation with mAbs or IVIG in 1% neonatal plasma. Phagocytosis of *S. epidermidis* N2297 by human neutrophils (MOI 10:1) in **(A)** a representative donor of 32-37 weeks GA and **(B)** a representative donor >37 weeks GA. FITC labelled bacteria were incubated in 1% neonatal plasma or 1% HI neonatal plasma supplemented with a concentration range of rF1, CR5133 or CR6453 IgG1 or IgG1 E345K or IVIG. Phagocytosis was quantified by flow cytometry and plotted as FITC GeoMFI of the neutrophils population. Data represent data of one independent experiment. Additional donors (n=5) can be viewed in **Figure S5**. GA, Gestational Age.

As complement activity increases with gestational age (12, 13), we divided the donors in two groups of different gestational age; 32-37 weeks GA (n=2) (**Figure 5A**) and >37 weeks GA (n=3) (**Figure 5B**). Similar to the results on strain ATCC 14990 (**Figure 3B**), rF1-IgG1 could induce phagocytosis and introduction of the hexamer-enhancing mutation in rF1-IgG1 did not result in beneficial effects. CR5133-IgG1 was also capable of inducing phagocytosis in presence of neonatal plasma and CR5133-IgG1 E345K further enhanced phagocytosis. CR6453-IgG1 could not induce phagocytosis, but introduction of the hexamer-enhancing mutation greatly enhanced phagocytosis. For all mAbs, the phagocytosis was completely dependent on the neonatal complement system, because when heat inactivated plasma was used, there was no phagocytosis (**Figure 5**, **S7**). We also compared mAb efficacy to IVIG efficacy. To observe a response in presence of complement, we added a maximum of 1 mg/mL IVIG in 1% plasma. However, this dose is ~10 times higher than clinically relevant, as neonatal clinical trials reach maximum IVIG concentrations of 7-15 mg/mL in 100% plasma (22, 43), which corresponds to 70-150 µg/mL in 1% plasma. In absence of complement, IVIG could not induce phagocytosis (**Figure S8**). For CR5133-IgG1 E345K, a concentration of only 0.042 µg/mL was needed to reach a comparable level of phagocytosis as 1000 µg/mL IVIG. For the other mAbs, a concentration of ~1 µg/mL mAb was needed to reach comparable levels to 1000 µg/mL IVIG. We did not observe differences between the two donor groups, thus mAbs can react with the neonate complement system of term (>37 weeks GA) and pre-term (32-37 weeks) infants. Concluding, we show complement activity in term and preterm infant plasma is sufficient to enhance IgG1 mediated opsonophagocytosis by human neutrophils.

## Discussion

*Staphylococcus epidermidis* is the most prevalent causative agent of late-onset sepsis in neonates, who are at high risk for infections because of transient immunodeficiency of immaturity (7–15, 44). In this study we show that monoclonal IgG1 antibodies against *S. epidermidis* can boost the neonatal complement system to opsonize bacteria for phagocytosis by neutrophils, which is an efficient way for the immune system to eliminate Gram-positive bacteria such as *S. epidermidis* (18).

Our study highlights that complement is essential for antibody-mediated phagocytosis of *S. epidermidis*. For the different IgG1 mAbs we compared, Fc gamma receptor mediated phagocytosis was absent (CR5133 and CR6453) or low (rF1). In the presence of complement, uptake was greatly enhanced. This is in agreement with previous work on *Streptococcus pneumoniae*, where IgG1 mAbs directed against capsule polysaccharide CPS6 were completely dependent on complement deposition (24). In contrast, we previously observed that naturally occurring IgG, IVIG (45) and a monoclonal antibody against WTA (23) can induce Fc receptor mediated phagocytosis of *S. aureus* in absence of complement. However, also in these studies, phagocytosis could be improved when complement was added.

Recently, Fc mutations and subclass switching to modify the interaction of a mAbs Fc-part with C1q to increase antibody-mediated complement deposition are being explored (46). This is because for optimal interaction with the six globular headpieces of C1q, target bound IgG molecules require organization into hexamers, which occurs *via* noncovalent Fc-Fc interactions (26, 27). Hexamer enhancing mutations have already been shown to enhance complement mediated lysis of *Neisseria gonorrhoeae* (47) and tumor cells (26) *via* the formation of membrane attack complex pores. Together with data on *S. aureus* (23) and *S. pneumoniae* (24), our study provides an important proof of concept that hexamer enhancing mutations can also potentiate opsonization and phagocytic killing of Gram-positive bacteria. This indicates that hexamer enhancing mutations could be applied to a broad range of pathogens and diseases.

Our data also indicates that introducing the hexamer enhancing E345K mutation can enhance mAbs to a similar extent or more than switching to IgG3 subclass. This is an important insight because IgG1 is established to be safe for antibody therapy in other fields than infectious diseases such as oncology and auto-immune diseases (48), is easier to produce and purify than IgG3 (46) and has a longer half-life than IgG3 (49).

This study also sheds light on the role of mAb epitopes in efficacy of phagocytosis. We here compared mAbs with an identical Fc backbone but different Fab domains on the same bacterial strain. Our data shows that the use of different Fab domains, which confers binding to epitopes, results in different efficacy between mAbs. This is novel compared to previous publications from our laboratory, in which we either compared mAbs recognizing different bacterial strains (*S. pneumoniae* (24)) or we studied one mAb (4497) with different Fc tails (*S. aureus* (23)). The direct comparison in this work is a strong indication that the epitope is crucial for the efficacy of mAbs to opsonize *S. epidermidis* for phagocytic killing. We hypothesize that the ideal epitope allows for IgG clustering, as literature describes that epitope density can influence hexamerization (26). Affinity for the epitope may also play a role, as binding with one Fab arm (instead of two) enhances hexamer formation (26, 50). In all, this stresses the importance of identifying the ideal epitope to target with mAb therapy.

Next to being able to trigger complement activation, for application of mAbs it is also important that they react with conserved antigens that are present on the majority of clinical isolates. This made rF1, although being the most potent mAb on our model strain, less ideal because it only bound ~50% of clinical isolates. Interestingly we showed that two mAbs (CR5133 and CR6453) could bind 19/20 clinical isolates. Even if CR5133- and CR6453-IgG1 were less potent in driving phagocytosis than rF1-IgG1, their functionality could be enhanced by hexamer enhancing mutations. The fact that we found two mAbs that bound 95% of clinical isolates indicates that mAb therapy for *S. epidermidis* holds potential, while for other pathogens, such as *S. pneumoniae*, the existence of many different serotypes will pose a challenge (51). The future of anti-bacterial preventive or therapeutic mAb therapies may well be in cocktails of multiple mAbs against different strains of a single species or against multiple species of interest (52).

Our study supports the presence of an effective complement system in neonates, enabling effective antibody induced opsonophagocytosis. Previous studies have reported a decreased complement activity and plasma concentrations of complement components in neonates, which is correlated with gestational age (11). Older studies described that most complement levels are at 50–70% of the adult values, rising to adult concentrations within 6 months after birth (11, 12, 15). Recent work describes significantly lower levels for approximately one third of the complement factors measured (28). We found lower complement activity compared to adults in the alternative pathway but not in the lectin and classical pathway. An explanation could be that we used r-Hirudin tubes to collect plasma in this study, which preserves complement activity the best (36). Even though alternative pathway activity, which is responsible for the complement amplification loop, was decreased, we showed that mAbs can greatly stimulate phagocytosis in the presence of neonatal complement. This confirms that neonatal complement can be activated by mAbs and that mAb therapy in neonates should be further explored.

As a proof of concept, we here show that mAbs (IgG1 and Fc : Fc enhanced IgG1) can react with the neonatal complement system to potentiate phagocytosis by healthy donor adult neutrophils. Others have shown neonatal neutrophils can efficiently phagocytose and kill *S. epidermidis* when opsonized with adult serum (44). Therefore, we expect that neonatal neutrophils will respond in the same manner as the adult neutrophils in our assay, although this will need further investigation.

In conclusion, we demonstrate that monoclonal antibodies against *S. epidermidis* can effectively induce opsonophagocytosis in the context of neonatal plasma. Opsonophagocytosis of *S. epidermidis* is dependent on complement activation and hexamer enhancing mutations in IgG effectuate more efficient opsonophagocytosis in neonatal plasma. Anti-*S. epidermidis* mAbs are a potential future preventive therapy that could be used in the neonatal setting to avoid *S. epidermidis* CLABSI in high-risk infants admitted to neonatal intensive care units.

## Data availability statement

Datasets are available on request: The raw data supporting the conclusions of this article will be made available by the authors, without undue reservation.

## Ethics statement

This study was reviewed and approved by Ethics Committee for Biobanking of the University Medical Center Utrecht. The patients/participants provided their written informed consent to participate in this study.

## Author contributions

Conception and design: LV, CB, KK, SR, MF. Sample preparation and collection of data: AF, LS, CB, CH, PA, FB. Analysis and interpretation of data: LV, CB, AF, KK. Contribution of reagents and tools: CH, PA, FB. Supervision: SR, KK, MF. Preparation of figures and tables: LV, AF. Collection of neonatal samples: CB, MF, MB, DV. Manuscript preparation: LV, AF. Revision of manuscript: CB, SR and MF. All authors read and approved the submitted version.

## Funding

This project was financially supported by a grant from the Wilhelmina Children’s Hospital Fund (MvdF) and by a PPP Allowance made available by Health~Holland (LSHM17026), Top Sector Life Sciences & Health, to stimulate public-private partnerships.

## Acknowledgments

The authors greatly thank J.T. van der Bruggen for providing *S. epidermidis* clinical isolates; prof. T. Mollnes and prof. P. Garred for providing clone Bh6 cells.

## Conflict of interest

FB is affiliated with Genmab.

The remaining authors declare that the research was conducted in the absence of any commercial or financial relationships that could be construed as a potential conflict of interest.

## Publisher’s note

All claims expressed in this article are solely those of the authors and do not necessarily represent those of their affiliated organizations, or those of the publisher, the editors and the reviewers. Any product that may be evaluated in this article, or claim that may be made by its manufacturer, is not guaranteed or endorsed by the publisher.
