# Supplementary material for: Monoclonal antibodies effectively potentiate complement activation and phagocytosis of Staphylococcus epidermidis in neonatal human plasma
Source: Front Immunol. 2022 Jul 29;13:933251. doi: 10.3389/fimmu.2022.933251 (PMC9372458; doi:10.3389/fimmu.2022.933251)
Supplement: Supplementary file 1 [file DataSheet_1.pdf]

Supplementary information

Supplementary Figures

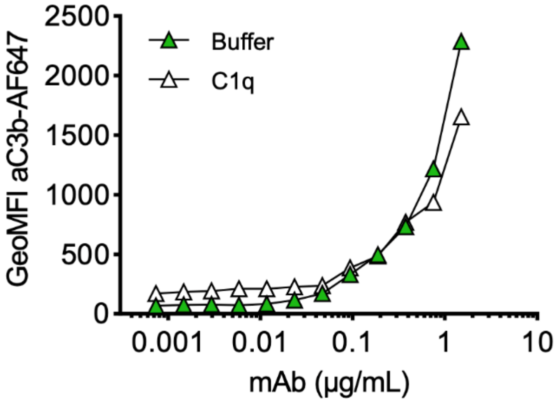

Figure S1. Effect of C1q supplementation in IgG/IgM depleted NHS on deposition of C3b. FITC labelled *S. epidermidis* ATCC 14990 were incubated in 1% ΔNHS supplemented with buffer or C1q and a concentration range of CR5133-IgG1. C3b deposition was detected by flow cytometry using an anti-neoC3b-AF647 antibody conjugate and plotted as AF647 GeoMFI of the FITC+ve bacterial population.

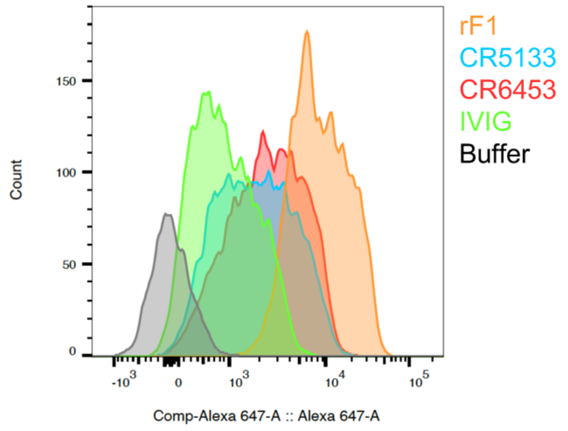

Figure S2. Flow cytometry histograms for Figure 1B. Representative of n=3.

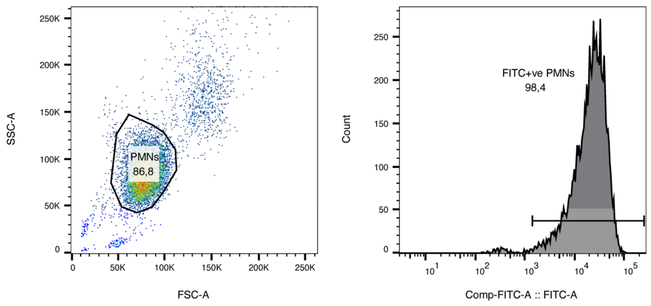

1 µg/mL rF1-IgG1 in 1% IgG/IgM dNHS

Ungated
10303

PMNs
8947

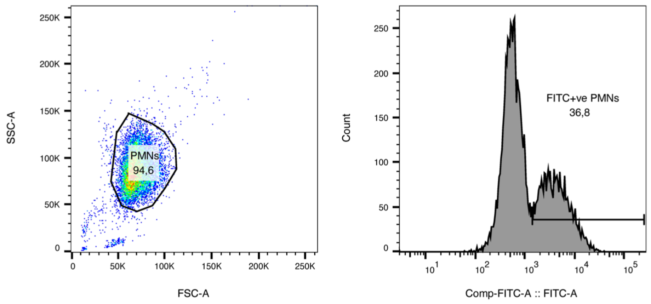

1 µg/mL CR5133-IgG1 in 1% IgG/IgM dNHS

Ungated
10228

PMNs
9677

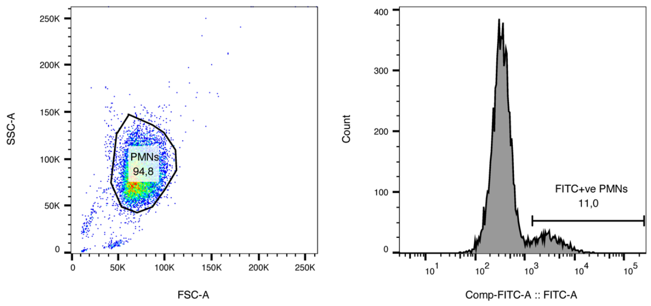

1 µg/mL CR6453-IgG1 in 1% IgG/IgM dNHS

Ungated
10236

PMNs
9704

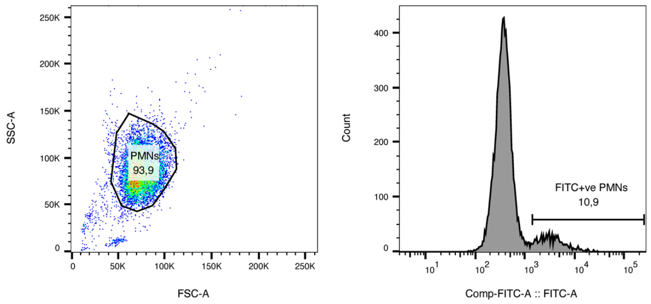

1 µg/mL aDNP-IgG1 in 1% IgG/IgM dNHS

Ungated
10243

PMNs
9617

Figure S3. Gating strategy for phagocytosis. Representative of n=3. A neutrophil population was gated based on FSC-SSC and GeoMFI of neutrophil in the FITC channel was analyzed.

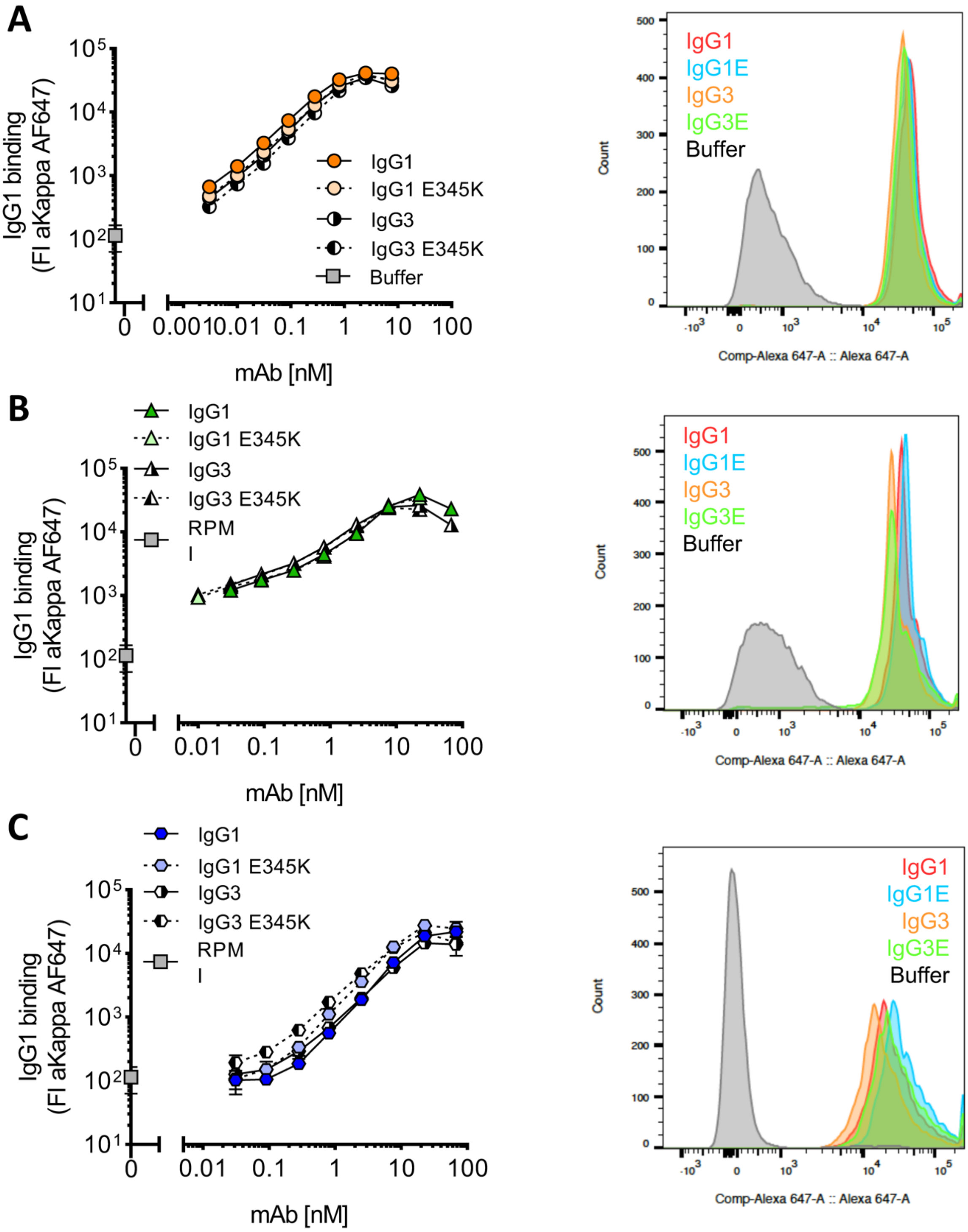

Figure S4. Titration of mAb variants. FITC labelled ATCC 14990 were incubated in a concentration range of rF1 **(A)**, CR5133 **(B)**, CR6453 **(C)** in IgG1, IgG1E, IgG3 or IgG3E variant. Flow cytometry histograms of 2.5 nM rF1, 22.7 nM CR5133 and 22.7 nM CR6453 are shown in the right panel. MAb binding detected with anti-kappa-AF647 and analyzed with flow cytometry. Data represent GeoMFI ± SD of three independent experiments.

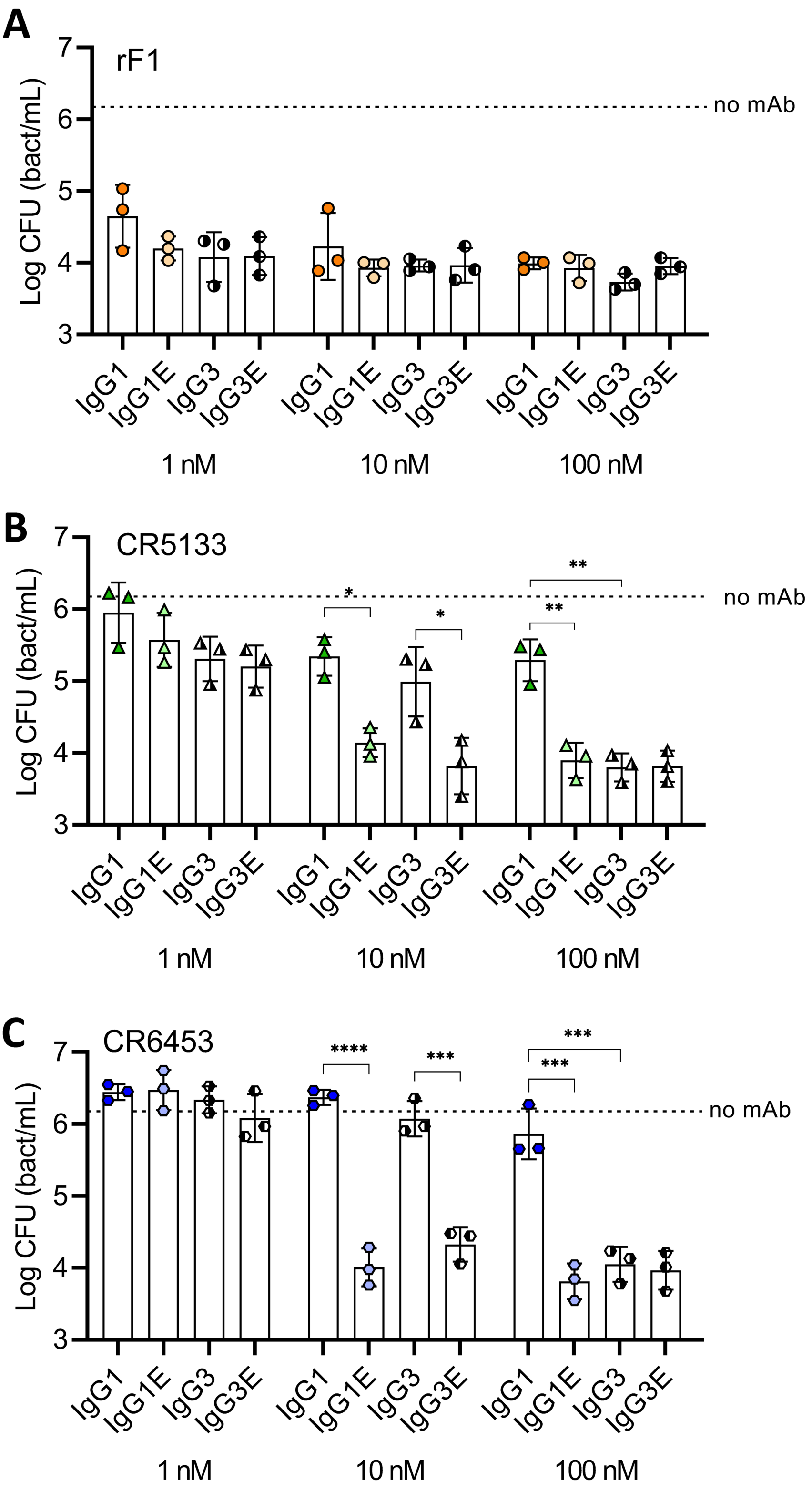

Figure S5. Killing at all concentrations of mAb variants tested. Killing of *S. epidermidis* ATCC 14990 by neutrophils (MOI 1:1) in presence of complement. Bacteria were incubated in 10% ΔNHS supplemented with different rF1 **(A)**, CR5133 **(B)**, CR6453 **(C)** concentrations. Bacterial survival was quantified after neutrophils lysis by serial dilution and CFU counting. Data represent mean ± SD of three independent experiments. One-way ANOVA followed by

Bonferroni correction was used to test the effect of mAb addition in ΔNHS, as well as the difference in bacterial survival of WT vs hexabody variant and the difference in effect between IgG1 and IgG3, *$P \leq 0.05$, **$P \leq 0.01$, ***$P \leq 0.001$ and ****$P \leq 0.0001$.

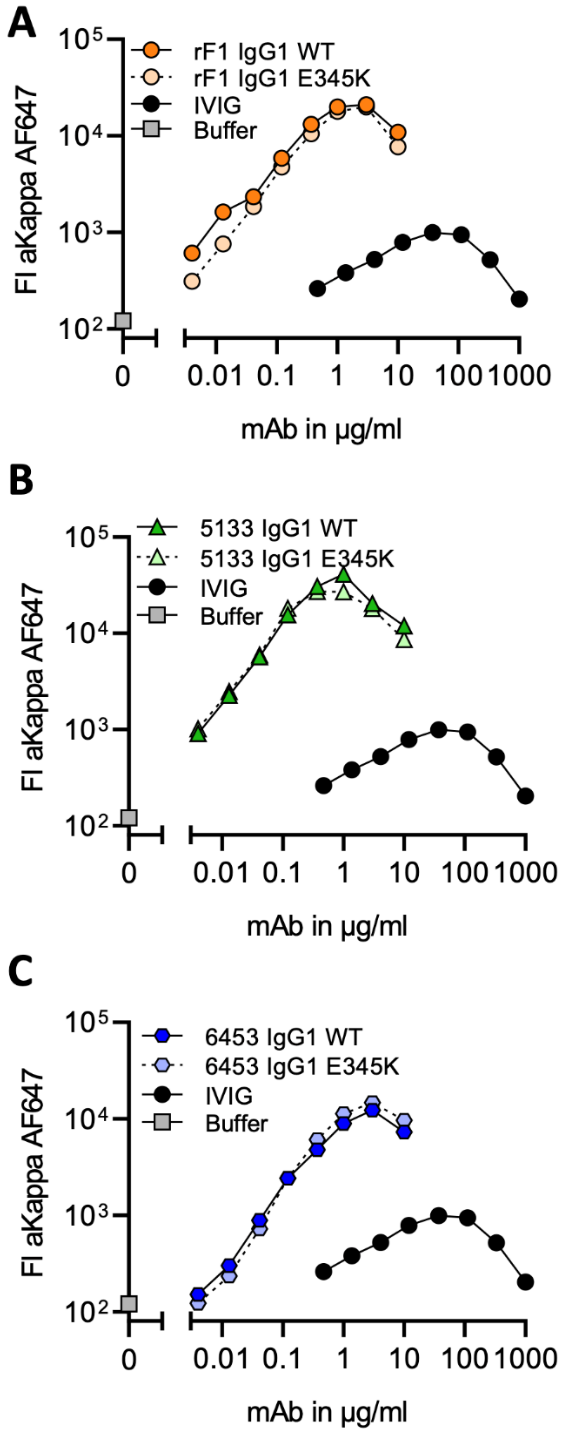

Figure S6. mAb binding to N2297. FITC labelled N2297 were incubated in a concentration range of IVIG, (A) rF1, (B) CR5133, (C) CR6453 IgG1. MAb binding detected with goat anti-hu-kappa-AF647 and analysed with flow cytometry. Data represent GeoMFI one experiment.

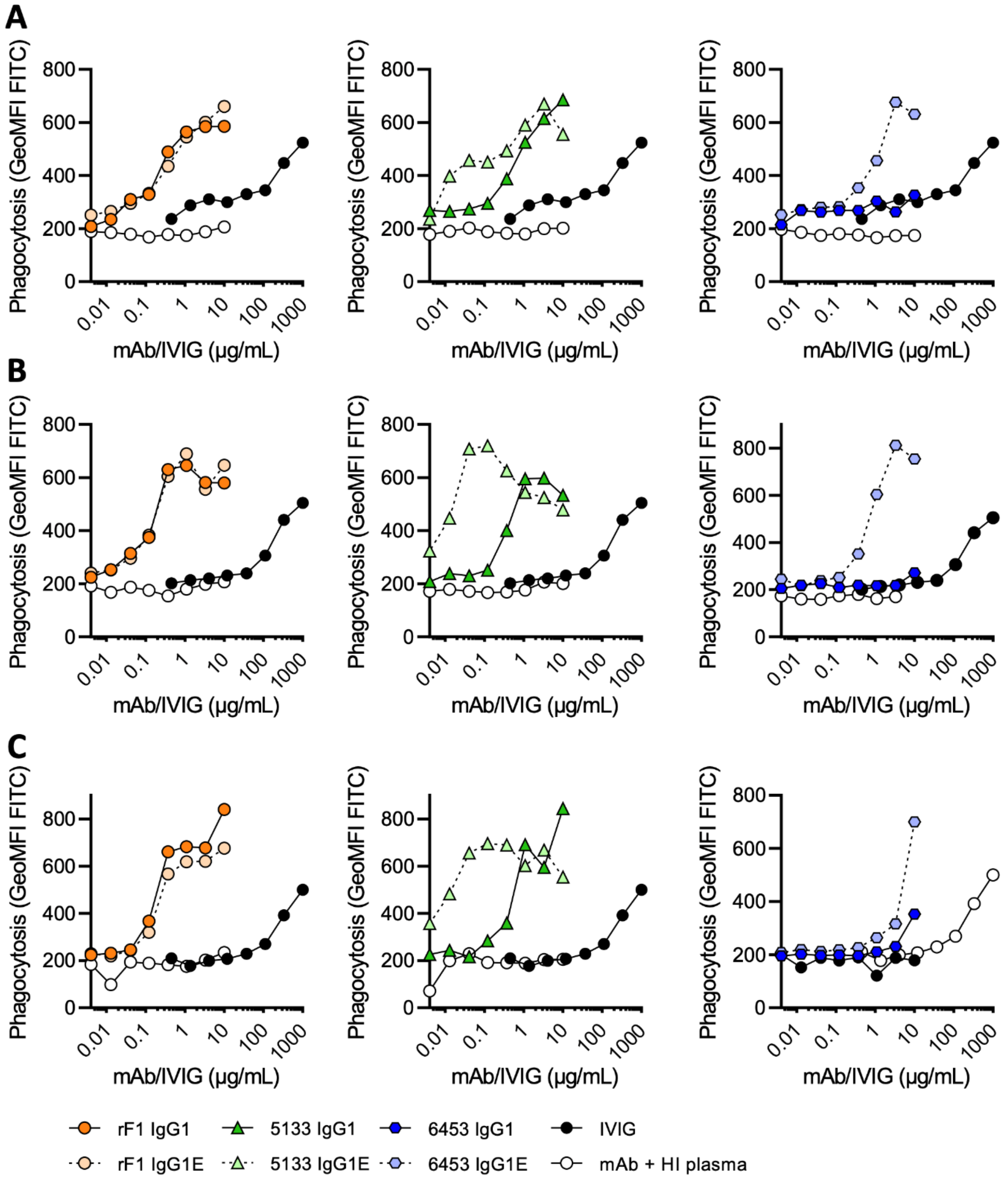

Figure S7. Phagocytosis of FITC labelled N2297 after incubation with mAbs or IVIG in 1% neonatal plasma. Phagocytosis of *S. epidermidis* N2297 by human neutrophils (MOI 10:1) in **(A)** a donor of 32-37 weeks GA and **(B, C)** 2 donors of >37 weeks GA. FITC labelled bacteria were incubated in 1% neonatal plasma or 1% HI neonatal plasma supplemented with a concentration range of rF1, CR5133 or CR6453 IgG1 or IgG1 E345K or IVIG. Phagocytosis was quantified by flow cytometry and plotted as FITC GeoMFI of the neutrophils population. Data represent data of one independent experiment. All donors (n=5) can be viewed in **Figure 5**. GA = Gestational Age

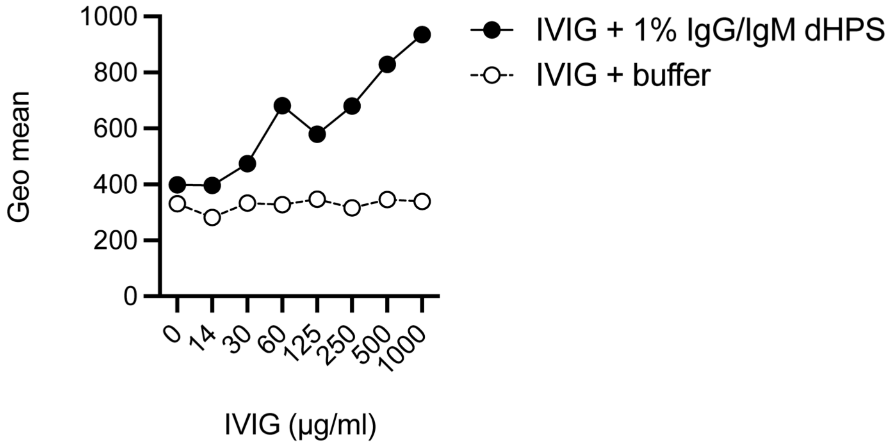

Figure S8. Phagocytosis of FITC labelled N2297 after incubation with mAbs or IVIG in 1% neonatal plasma. Phagocytosis of *S. epidermidis* N2297 by human neutrophils (MOI 10:1). FITC labelled bacteria were incubated in 1% IgG/IgM depleted NHS or buffer supplemented with a concentration range of IVIG. Phagocytosis was quantified by flow cytometry and plotted as FITC GeoMFI of the neutrophils population. Data represent data of one independent experiment.

Supplementary Tables

Table S1. Protein sequences used for human monoclonal antibody production.

Variable and constant heavy chain and light chain amino acid sequences used for antibody production. Residues E345K are highlighted in yellow. All antibodies were of kappa class, except otherwise indicated.

**Antibody clone**	**Sequence**	**Patent number or reference**
**VH: variable region heavy chain**		
rF1	EVQLVESGGGLVQPGGSLRLSCAASGFTLSRFAMS WVRQAPGRGLEWVASINSGNNPYYARSVQYRFTV SRDVSQNTVSLQMNNLRAEDSATYFCAKDHPSSG WPTFDSWGPGTLVTVSS	WO/2016/090040 A1 (1)
M130	QVQLQQSGPGILQPSQTLSLTCSFSGFSLSTSGMSVS WIRQPSGKGLEWLAHIFWDDDKRYNPSLKSRLTVS KDTSSNQVFLKITSVGTADTATYYCARNYDYDWF VYWGQGTLVTVSA	US 2002/041033 (2)
CR5132	EVLESGGGLVQPGGSLRLSCSDSGFSFNNYWMTW VRQAPGKGLEWVANINRDGSDKYHVDSVEGRFTIS RDNSKNSLYLQMNNLRADDAA VYFCARGGRTTSWYWRNWGQGTLVTVSS	US 2012/0141493 A1 (3)
CR5133	EVQLVETGGGLVKPGGSLRLSCSASRFSFRDYYMT WIRQAPGKGPEWVSHISGSGSTIYYADSVRGRFTIS RDNAKSSLYLQMDSLQADDTAVYYCARGGRATSY YWVHWGPGTLVTVSS	US 2012/0141493 A1 (3)
CR6453	EVQLVESGGGLVQPGGSLRVSCAASGFTFSSYWMT WVRQAPGKGLEWVANIKKDGSEKYYVDSVKGRFS ISRDNAKDSLYLQMSSLRAEDTAVYYCARGGSSSS FYWWLWGKGTTVTVSS	US 2012/0141493 A1 (3)
A120	EVMLVESGGGLVQPKGSLKLSCAASGFTFNTYAM NWVRQAPGKGLEWVARIRSKSNNYATYYADSVK DRFTISRDDSQSMLYLQMNNLKTEDTAMYYCVRR GGKETDYAMDYWGQGTSVTVSS	WO/03/059259 A2 (4)
CR6166 (Lambda)	QVQLVQSGAEVKKPGESLKISCKGSGYSFTSYWIG WVRQMPGKGLEWMGIIYPGDSDTRYSPSFQGQVTI SADKSISTAYLQWSSLKASDTAMYYCARRASIVGA THFDYWGQGTLVTVSS	US 2012/0141493 A1 (3)
CR6171	EVQLVETGGVAVQPGRSLRLSCAASGFSFRDYGMH WVRQAAGKGLEWVAFIWPHGVNRFYADSMEGRF TISRDDSKNMLYLEMNNLRTEDTALYYCTRDQDY VPRKYFDLWGRGTLVTVSS	US 2012/0141493 A1 (3)

CR6176	VQLQESGPRLVKPSETLSLTCNVSDDSITSYGYYWG WIRQPPGEALEWIGNVFYSGMAYYNPSLKSRVTILI DTSKKQFSLRLNSVTAADTAIYYCARVPFLMFRVKI VQGTGAFDIWGQGTMVTVSS	US 2012/0141493 A1 (3)
G2a2	DVRLQESGPGLVKPSQSLSLTCSVTGYSITNSYYWN WIRQFPGNKLEWMVYIGYDGSNNYNPSLKNRISIT RDTSKNQFFLKLNSVTTEDTATYYCARATYYGNYR GFAYWGQGTLVTVSA	(5)
**VL: variable region light chain**		
rF1	DIQLTQSPSALPASVGDRVSITCRASENVGDWLAW YRQKPGKAPNLLIYKTSILESGVPSRFSGSGSGTEFT LTISSLQPDDFATYYCQHYMRFPYTFGQGTKVEIK	WO/2016/090040 A1 (1)
M130	DIKMTQSPLTLSVTIGQPASISCKSSQSLLDSDGKTY LNWLLQRPGQSPKRLIYLVSKLDSGVPDRFAGSGS GTDFTLKISRVEAEDLGVYYCWQGTHFPLTFGAGT KLELK	US 2002/041033 (2)
CR5132	STDIQMTQSPSTLSASVGDRVTITCRASQSISSWLA WYQQKPGKAPKLLIYKASSLESGVPSRFSGSGSGTE FTLTISSLQPDDFATYYC QQYNSYPLTFGGGTKLEIK	US 2012/0141493 A1 (3)
CR5133	STEIVLTQSPATLSLSPGERATLSCRASQSVSGYLG WYQQKPGQAPRLLIYGASSRATGIPDRFSGSGSGTD FTLTISRLEPEDFAVYYCQQYGSSPLTFGGGTKLEIK	US 2012/0141493 A1 (3)
CR6453	EIVLTQSPGTLSLSPGERATLSCRASQSVSSNYLAW YQQKPGQAPRLLVYGASSRATGIPDRFSGSGSGTDF TLTISRLEPEDFAVYHCQQYAGSPWTFGQGTKVEIK	US 2012/0141493 A1 (3)
A120	DIVLSQSPAILSASPGEKVTMTCRASSSVSYMHWY QQKPGSSPKPWIYATSNLASGVPARFSGSGSGTSYS LTISRVEAEDAATYYCQQW SSNPPTFGGGTKLEIK	WO/03/059259 A2 (4)
CR6166 (Lambda)	QSALTQPPSASGSPGQSVTISCTGTSSDVGGYNYVS WYQQHPGKAPKLMIYEVSKRPSGVPDRFSGSKSGN TASLTVSGLQAEDEADYYCSSYAGSNNLVFGGGTK LTVLG	US 2012/0141493 A1 (3)
CR6171 (Lambda)	QSVLTQPPSLSVSPGQTASISCSGDKLGDKYVSWYQ QRPGQSPVLVIYHDTKRPSGIPERFSGTNSGNTATLT ISGTQILDEADYYCQVWDRSTVVFGGGTQLTVL	US 2012/0141493 A1 (3)
CR6176	EIVLTQSPGTLSLSPGERATLSCRASQSVSSSYLAW YQQKPGQAPRLLIYGASSRATGIPDRFSGSGSGTDF TLTISSLEPEDFAVYYCQQYGSSSITFGQGTRLEIK	US 2012/0141493 A1 (3)

G2a2	DIRMTQTTSSLSASLGDRVTISCRASQDISNYLNWY<br>QQKPDGTVKLLIYYTSRLHSGVPSRFSGSGSGTDYS<br>LTISNLEQEDIATYFCQQGNTLPWTFGGGTKLEIK	(5)
**CH: constant regions heavy chain**		
IgG1	ASTKGPSVFPLAPSSKSTSGGTAALGCLVKDYFPEP<br>VTVSWNSGALTSGVHTFPAVLQSSGLYSLSSVVTV<br>PSSSLGTQTYICNVNHKPSNTKVDKKVEPKSCDKT<br>HTCPPCPAPELLGGPSVFLFPPKPKDTLMISRTPEVT<br>CVVVDVSHEDPEVKFNWYVDGVEVHNAKTKPREE<br>QYNSTYRVVSVLTVLHQDWLNGKEYKCKVSNKAL<br>PAPIEKTISKAKGQPREPQVYTLPPSREEMTKNQVS<br>LTCLVKGFYPSDIAVEWESNGQPENNYKTTPPVLD<br>SDGSFFLYSKLTVDKSRWQQGNVFSCSVMHEALH<br>NHYTQKSLSLSPGK	(6)<br>(7)
IgG3	ASTKGPSVFPLAPCSRSTSGGTAALGCLVKDYFPEP<br>VTVSWNSGALTSGVHTFPAVLQSSGLYSLSSVVTV<br>PSSSLGTQTYTCNVNHKPSNTKVDKRVELKTPLGD<br>TTHTCPRCPEPKSCDTPPPCPRCPEPKSCDTPPPCPR<br>CPEPKSCDTPPPCPRCPAPELLGGPSVFLFPPKPKDT<br>LMISRTPEVTCVVVDVSHEDPEVQFKWYVDGVEV<br>HNAKTKPREEQYNSTFRVVSVLTVLHQDWLNGKE<br>YKCKVSNKALPAPIEKTISKTKGQPREPQVYTLPPS<br>REEMTKNQVSLTCLVKGFYPSDIAVEWESSGQPEN<br>NYNTTPPMLDSDGSFFLYSKLTVDKSRWQQGNIFS<br>CSVMHEALHNRFTQKSLSLSPGK	(6)<br>(7)
**CL: constant regions light chain**		
Kappa class	RTVAAPSVFIFPPSDEQLKSGTASVVCLLNNFYPRE<br>AKVQWKVDNALQSGNSQESVTEQDSKDSTYSLSST<br>LTLSKADYEKHKVYACEVTHQGLSSPVTKSFNRGE<br>C	(7)
Lambda class	GQPKAAPSVTLFPPSSEELQANKATLVCLISDFYPG<br>AVTVAWKADSSPVKAGVETTTPSKQSNNKYAASS<br>YLSLTPEQWKSHRSYSCQVTHEGSTVEKTVAPTEC<br>S	(7)

Supplementary references

1. Hazenbos WLW, Kajihara KK, Vandlen R, Morisaki JH, Lehar SM, Kwakkenbos MJ, et al. Novel Staphylococcal Glycosyltransferases SdgA and SdgB Mediate Immunogenicity and Protection of Virulence-Associated Cell Wall Proteins. PLoS Pathog. 2013;9(10).
2. Schuman RF, Kokai-kun JF, Foster SJ, Stinson JR, Fischer GW. Multifunctional monoclonal antibodies directed to peptidoglycan of gram-positive bacteria. 2007.
3. Throsby M, Geuijen C, Kruif C. Human binding molecules having activity against staphylococci and uses thereof. 2012.
4. Stinson JR, Schuman RF, Mond JJ, Lees A, Fischer GW. Opsonic monoclonal and chimeric antibodies specific for lipoteichoic acid of Gram positive bacteria. 2007.
5. Gonzalez ML, Frank MB, Ramsland PA, Hanas JS, Waxman FJ. Structural analysis of IgG2A monoclonal antibodies in relation to complement deposition and renal immune complex deposition. Mol Immunol. 2003;40(6):307–17.
6. Cruz AR, den Boer MA, Strasser J, Zwarthoff SA, Beurskens FJ, de Haas CJC, et al. Staphylococcal protein A inhibits complement activation by interfering with IgG hexamer formation. Proc Natl Acad Sci U S A. 2021;118(7).
7. Kabat EA, Wu TT, Perry HM, Gottesman KS, Foeller C. Sequences of proteins of immunological interest. Vol. 138, Analytical Biochemistry. 1984. 265 p.
